# Fas Ligand localizes to intraluminal vesicles within NK cell cytolytic granules and is enriched at the immune synapse

**DOI:** 10.1002/iid3.219

**Published:** 2018-04-11

**Authors:** Jeansun Lee, Nele M.G. Dieckmann, James R. Edgar, Gillian M. Griffiths, Richard M. Siegel

**Affiliations:** ^1^ CIMR, Department of Medicine Cambridge University Cambridge UK; ^2^ Immunoregulation Section, Autoimmunity Branch, National Institutes of Arthritis and Musculoskeletal and Skin Diseases National Institutes of Health Bethesda Maryland USA

**Keywords:** Cytolytic granules, fas ligand (CD178), immune synapse, intra‐luminal vesicles (ILVs)

## Abstract

**Introduction:**

T cell and NK cell cytotoxicity can be mediated via the perforin/granzyme system and Fas Ligand (FasL, CD178). FasL is synthesized as a type II transmembrane protein that binds its cognate receptor Fas (CD95). Membrane‐bound FasL is expressed on the plasma membrane of activated lymphocytes and is the main form of FasL with cytotoxic activity, but whether FasL is delivered to the immune synapse along with granzyme and perforin‐containing granules is unclear.

**Methods:**

We stably expressed FasL‐fluorescent fusion proteins into human NK cells and examined the localization of FasL relative to other intracellular markers by confocal and immunoelectron microscopy, and examined the trafficking of FasL during formation of immune synapses with HLA‐deficient B cells.

**Results:**

FasL co‐localized with CD63 more strongly than perforin or Lamp1+ in cytolytic granules. Electron microscopy revealed that FasL is enriched on intraluminal vesicles (ILVs) adjacent to the dense‐core within cytolytic granules. In NK cells forming immune synapses with HLA‐deficient B cells, a portion of FasL‐containing granules re‐localize toward the immune synapse, while a distinct pool of FasL remains at the distal pole of the cell.

**Conclusions:**

Localization of FasL to intra‐luminal vesicles within cytolytic granules facilitates FasL trafficking to immune synapses and cytotoxic function in NK cells.

## Introduction

Natural killer (NK) and cytotoxic CD8^+^ T lymphocytes (CTL) kill virus‐infected and tumor cells through the targeted delivery of cytotoxic granules. These cells can induce target cell death though two major mechanisms. One involves the rapid release of lytic granules containing cytotoxic molecules including perforin and granzymes into a synaptic cleft formed between the effector and target cell upon triggering of the TCR or NK cell activating receptors. Upon perforin‐facilitated entry into the target cell, granzyme protease activity cleaves critical cellular substrates leading to rapid DNA degradation and cell death. The second mechanism involves interactions between the TNF‐family cytokine Fas ligand (FasL, CD178) on effector cells with its cognate receptor Fas (CD95) on the cell surface of target cells. The binding of membrane‐bound FasL with Fas induces cell death during target cell killing as well as autocrine and paracrine T cell death 1. Although the perforin/granzyme system mediates efficient target cell killing, both CTL and NK cells appear to use FasL to some extent to kill target cells. In CTL, deficiencies in either the perforin‐mediated or Fas‐FasL pathway impairs, but does not completely abolish the killing ability of CTLs, whereas deficiencies of both systems abolish CTL activity 2, 3. In NK cells, cytolytic activity can also be significantly inhibited in the presence of antibodies against Fas, suggesting a non‐redundant role for FasL‐Fas interactions in NK cell‐mediated cytotoxicity as well 4, 5. Thus, the FasL‐Fas pathway appears to be an alternate lytic system to the perforin/granzyme pathway in both CTL and NK cells.

Genetic studies have revealed that Fas‐FasL interactions are important in maintaining lymphocyte homeostasis and immunological self‐tolerance, as seen in the lympho‐accumulation and autoantibody formation that ensues from deficiency in either Fas or FasL in *lpr* and *gld* mice 6, 7. In humans, dominant negative mutations in Fas or FasL cause most cases of the Autoimmune Lymphoproliferative Syndrome (ALPS) 8, 9. FasL‐Fas interactions also play a role in killing virally infected cells and maintaining homeostasis of the anti‐viral CTL pool during chronic infection 10, 11, 12. These studies highlight the importance of understanding the mechanism by which FasL kills target cells.

FasL is synthesized as a type II transmembrane protein and can be secreted in soluble form after cleavage of the extracellular Fas‐binding domain by the metalloprotease ADAM10 13. However, soluble FasL does not exert cytotoxicity and may in some cases inhibit apoptosis induced by membrane‐bound FasL 14. Membrane‐bound surface FasL increases significantly upon T cell activation 15, 16, and FasL at the plasma membrane may be capable of exerting lytic functions that would not require conjugate formation and directional granule release. Additionally, FasL has been detected on the membranes of secreted microvesicles, which are thought to derive from the inner vesicles of multivesicular bodies (MVB). Vesicular FasL may also be cytotoxic 17, 18, 19, 20, but would require directed secretion into the immunological synapse for efficient function. Which of these forms of membrane FasL exerts cytotoxicity, and whether FasL traffics to the immunological synapse upon TCR and NK cell receptor triggering, is not known.

To better understand the mechanisms by which NK cells use FasL to kill target cells, we studied the intracellular localization of FasL in human NK cells virally transduced with FasL‐fluorescent fusion proteins. We found that FasL is enriched on the membranes of intraluminal vesicles (ILVs) within CD63 and LAMP1‐expressing secretory lysosomes, but is not found in the dense cores of secretory lysosomes, where perforin is present. FasL‐containing secretory lysosomes accumulated at the immune synapse during NK‐cell recognition of MHC‐deficient target cells, with a secondary pool of FasL vesicles accumulating at the pole opposite to the immune synapse. These studies identify vesicular FasL as the form of FasL that is delivered to the immune synapse, mediating its cytotoxic potential.

## Results

### FasL localizes to a secretory lysosome compartment distinct from perforin in human NK cells

Previous studies in transiently transfected RBL cells and native FasL in fixed T and NK cells have shown that FasL primarily localizes in secretory lysosomes with a minority of FasL on the cell surface 21. To examine FasL trafficking in living human NK cells, we generated clones of the human NK cell line YT that were retrovirally transduced to stably express a chimeric FasL protein with GFP fused to its intracellular amino terminus. To prevent spontaneous cytotoxic activity of FasL, the Fas‐binding sequence of the extracellular domain of FasL (amino acid residue 218) was altered with a point mutation derived from *gld* mice, rendering FasL non‐functional 22, 23, 24. YT clones expressing FasL‐GFP at equivalent levels were selected for study.

**Figure 1:**
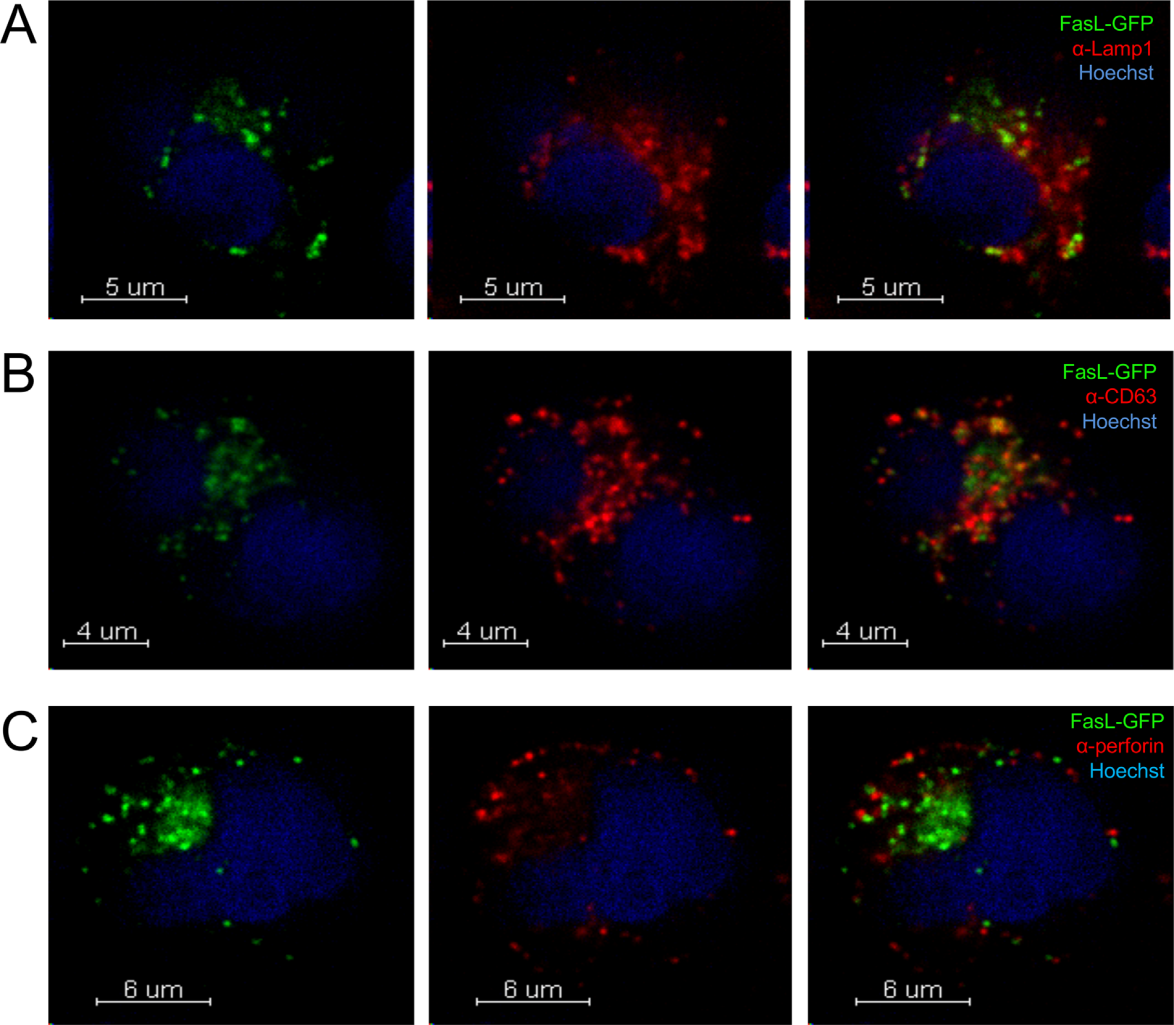
FasL in NK cells predominantly colocalizes to CD63^+^ vesicles distinct from LAMP1 and perforin. Confocal immunofluorescence microscopy (mid‐cell slices) of YT cells stably expressing FasL‐GFP (green) were stained with anti‐LAMP1 antibody to label LAMP1^+^ compartments (red) (A), anti‐CD63 antibody to label CD63+ compartments (red) (B), and with anti‐perforin antibody (δG9) to label perforin‐containing compartments (red) (C). The nuclei of YT cells were labeled with Hoechst dye (blue).

**Figure 2:**
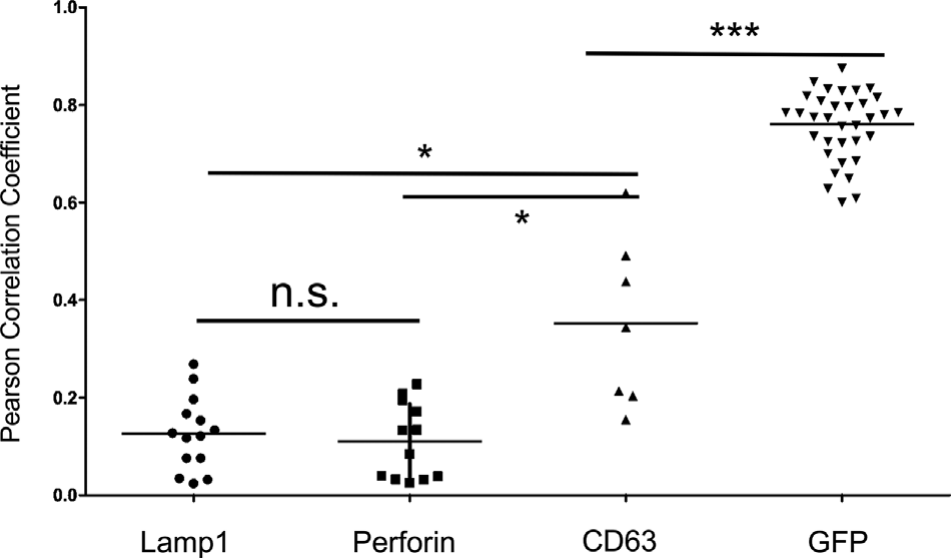
Quantitation of FasL colocalization with vesicle markers. The Pearson coefficient of FasL colocalization with LAMP1, perforin, CD63, and GFP is shown, with each point representing colocalization within an image stack. Asterisks indicate statistically significant colocalization differences for FasL with the indicated marker via Mann‐Whitney test (two‐tailed, 95% confidence intervals), with CD63 vs LAMP1: *p* = 0.025(*); GFP‐FasL to CD63 vs GFP‐FasL to anti‐GFP: *p* < 0.0001(***); and LAMP1 versus Perforin: nonsignificant, *p* = 0.817 (n.s).

We first examined FasL localization in YT cell clones expressing FasL‐GFP using confocal microscopy. Immuno‐stained FasL and GFP showed complete colocalization, validating GFP as a marker for FasL (Fig. S1). Using antibodies labeling CD63, LAMP1, and perforin‐positive compartments in NK cells, we studied the subcellular localization of FasL relative to components of the secretory lysosome. LAMP1 is a protein found on the limiting membrane of lysosomes, while CD63 is a protein found both on the limiting membrane and on the internal membranes of MVBs. In contrast, perforin and granzymes are often found in the dense core of secretory lysosomes 27, 28, 29. We observed the greatest overlap between FasL and CD63+ compartments, with reduced colocalization with LAMP1 or perforin (Fig. 1). Quantitative image analysis of colocalization in multiple cells confirmed this hierarchy, with greater colocalization of FasL with CD63 than with LAMP1 or perforin (Fig. 2).

In addition we analyzed cells using a “line‐tool” method to visualize the degree of co‐localization between FasL and secretory compartments. This method quantitates the peak overlaps that occur between FasL and immunostained granules along a line selected to go through multiple granules in a single optical slice of the cell (Fig. S2). Concordant with the colocalization analysis shown in Figure 2, there was more frequent overlap in the peaks between FasL and CD63 than with perforin or LAMP1, although FasL was frequently observed in close proximity perforin, suggesting that they may in distinct compartments within the secretory lysosome. Taken together, these data show that in lymphocytes before contact with a target cell, FasL is enriched in CD63^+^ vesicles and adjacent, but not colocalized with perforin within granules.

**Figure 3:**
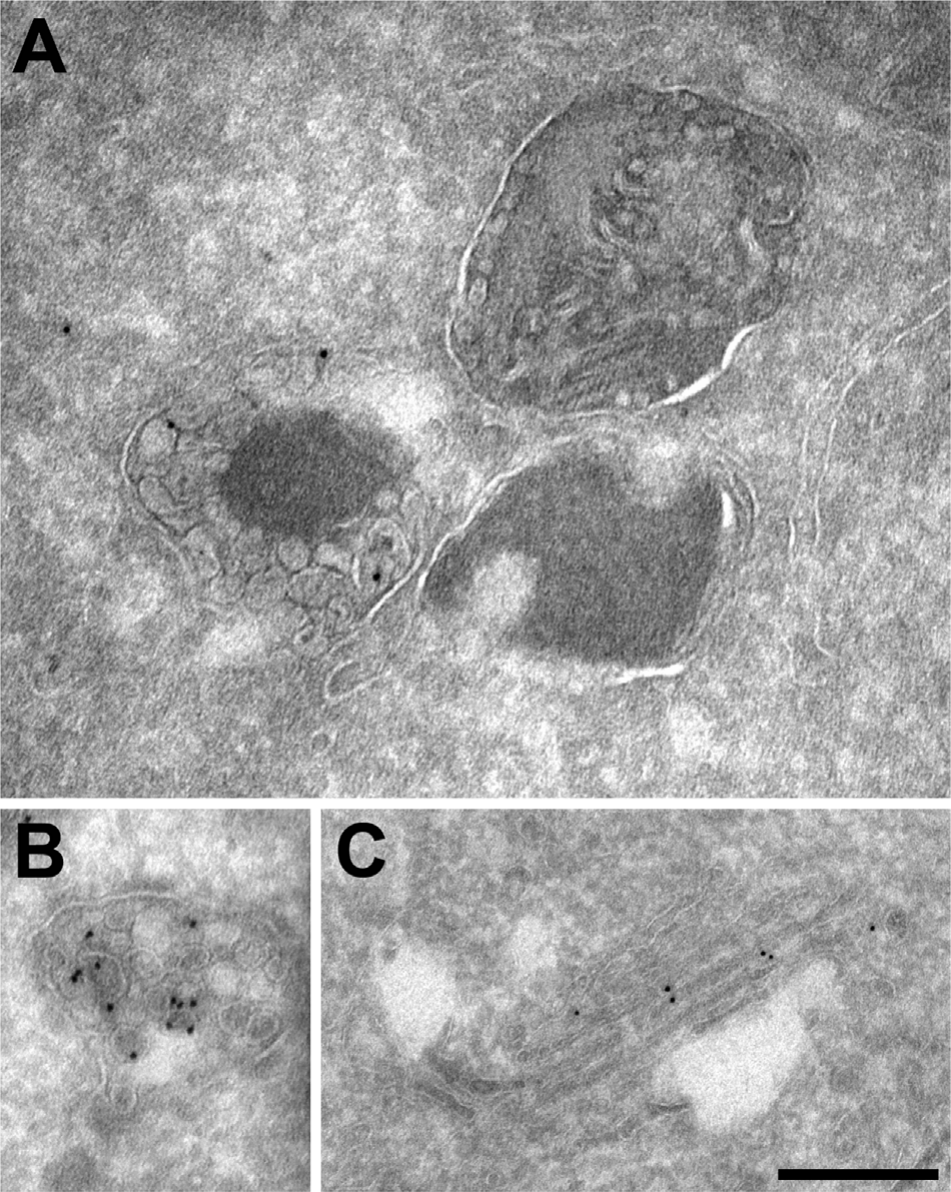
Ultrastructural analysis of FasL localization in YT cells. Representative electron microscopy images of immunogold‐labeled cryo‐sections from stable YT cells expressing GFP‐tagged FasL, visualized with anti‐GFP and protein‐A gold particles. (A) Shows the electron dense core and intraluminal vesicles of cytolytic granules bound by an outer limiting membrane, with FasL gold particle labeling exclusively in the intraluminal vesicles and not the dense core. (B) shows an MVB that contains only ILVs with prominent immunogold labeling. In (C) GFP‐FasL labeling is shown within the Golgi stack. Scale bars = 200 nm.

To investigate the localization of FasL in secretory vesicles in relation to perforin and granzyme B in more detail, we used immunogold labeling with an antibody against GFP to localize the FasL‐GFP fusion protein via electron microscopy (EM). In YT cells stably expressing FasL‐GFP, the GFP signal was detected predominantly on the ILVs within the secretory lysosomes, but was excluded from the electron dense cores which contain perforin and granzymes (Fig. 3A,B). The number of gold particles per intraluminal vesicle (ILV) carrying FasL, ranged from 1 to 12 gold particles per ILV (mean = 3, *n* = 66) (Fig. 3B). In addition, we detected minor gold labeling FasL‐GFP in the Golgi (Fig. 3C). These data show that FasL is sorted to the ILVs of secretory lysosomes, positioning FasL to be present on the surface of a secreted vesicle in membrane‐bound form, whereas FasL was not present in the electron‐dense cytolytic granules

### FasL relocalizes toward the immune synapse upon target cell recognition

**Figure 4:**
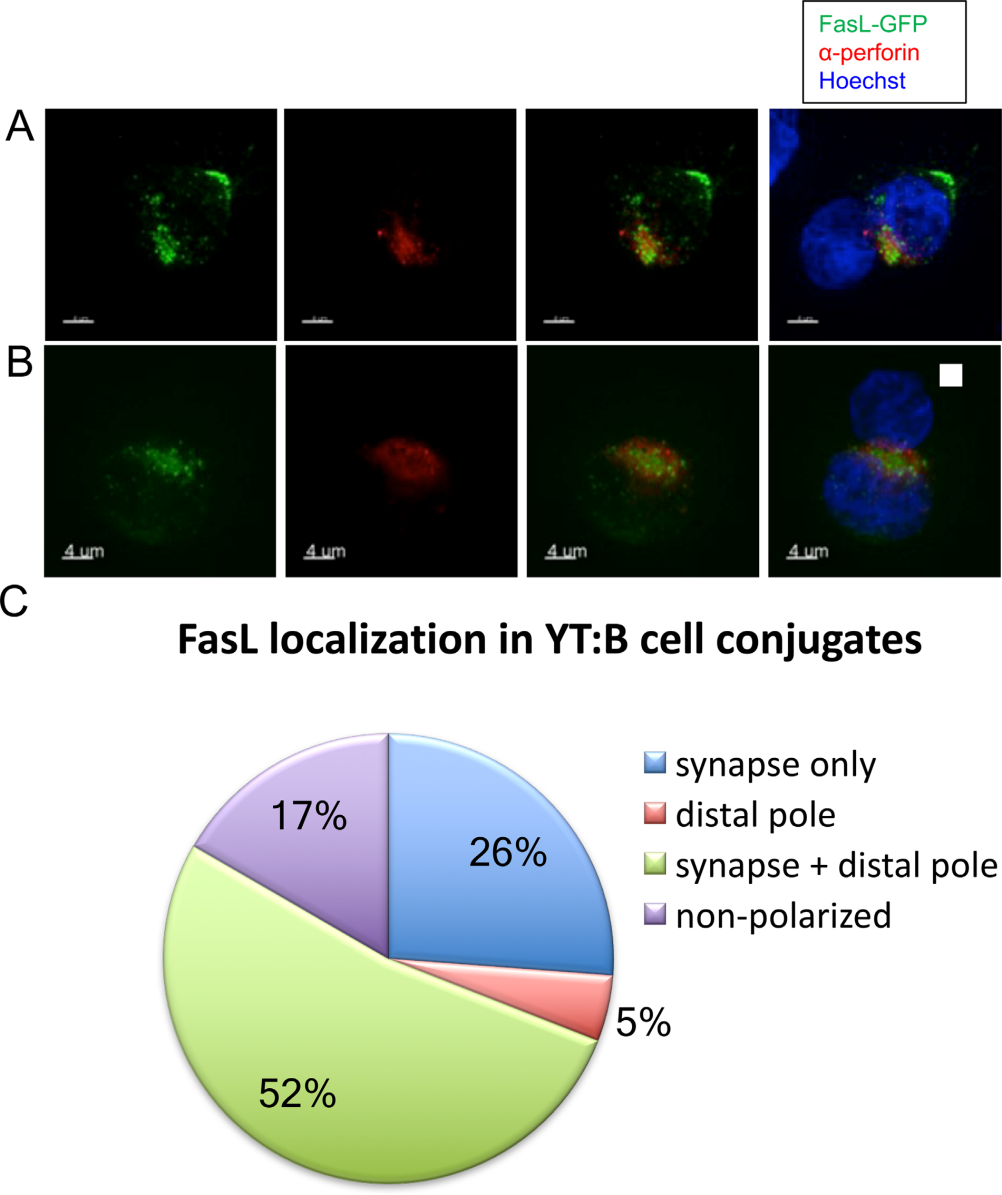
Distinct patterns of FasL subcellular localization during immune synapse formation. (A) and (B) Confocal immunofluorescence microscopy of YT cells stably expressing FasL‐GFP (green) in conjugates with target HLA class I‐deficient 721.221 B cells were labeled with anti‐perforin (δG9) to mark perforin granules (red). Examples of FasL localized to the synapse and distal pole (A) and synapse exclusively (B) are shown. The nuclei of target B cells were pre‐labeled with Coumarin‐blue dye. All images were taken 0–12 min after YT cells came into contact with 721.221 B cells. (C) Quantification (percentages) of YT:B cell conjugates (*n* = 42) that displayed FasL localization at the distal pole, immune synapse, or both.

To examine the trafficking behavior of FasL during interactions between NK and target cells, we imaged FasL‐GFP in YT cells that had formed conjugates with 721.221 cells, a human B cell line lacking MHC class I expression. The lack of MHC class I relieves signaling through inhibitory receptors on NK cells, enabling conjugate formation, NK cell activation and target cell killing. We identified YT cells that had formed immune synapses with target cells as those in which perforin re‐localized to the interface with the target cell. Confocal images of YT cells in which endogenous perforin had re‐localized to the site of contact with 721.221 B cells revealed three distinct patterns of FasL‐GFP localization (Fig. 4C). In 52% of conjugates analyzed (*n* = 42), intracellular FasL‐containing vesicles polarized toward the immunological synapse and colocalized with perforin, with some FasL remaining at the distal pole of the cell (Fig. 4A). In other conjugates (26%), almost all FasL was proximal to the immunological synapse (Fig. 4B). FasL was exclusively localized to the distal pole of the cell only in 5% of conjugates examined. These results suggest that FasL is delivered to the immunological synapse after target cell recognition, but less efficiently than perforin, with a significant percentage of cells retaining FasL‐containing vesicles at the opposite pole of the cell, distal from the immune synapse.

**Figure 5:**
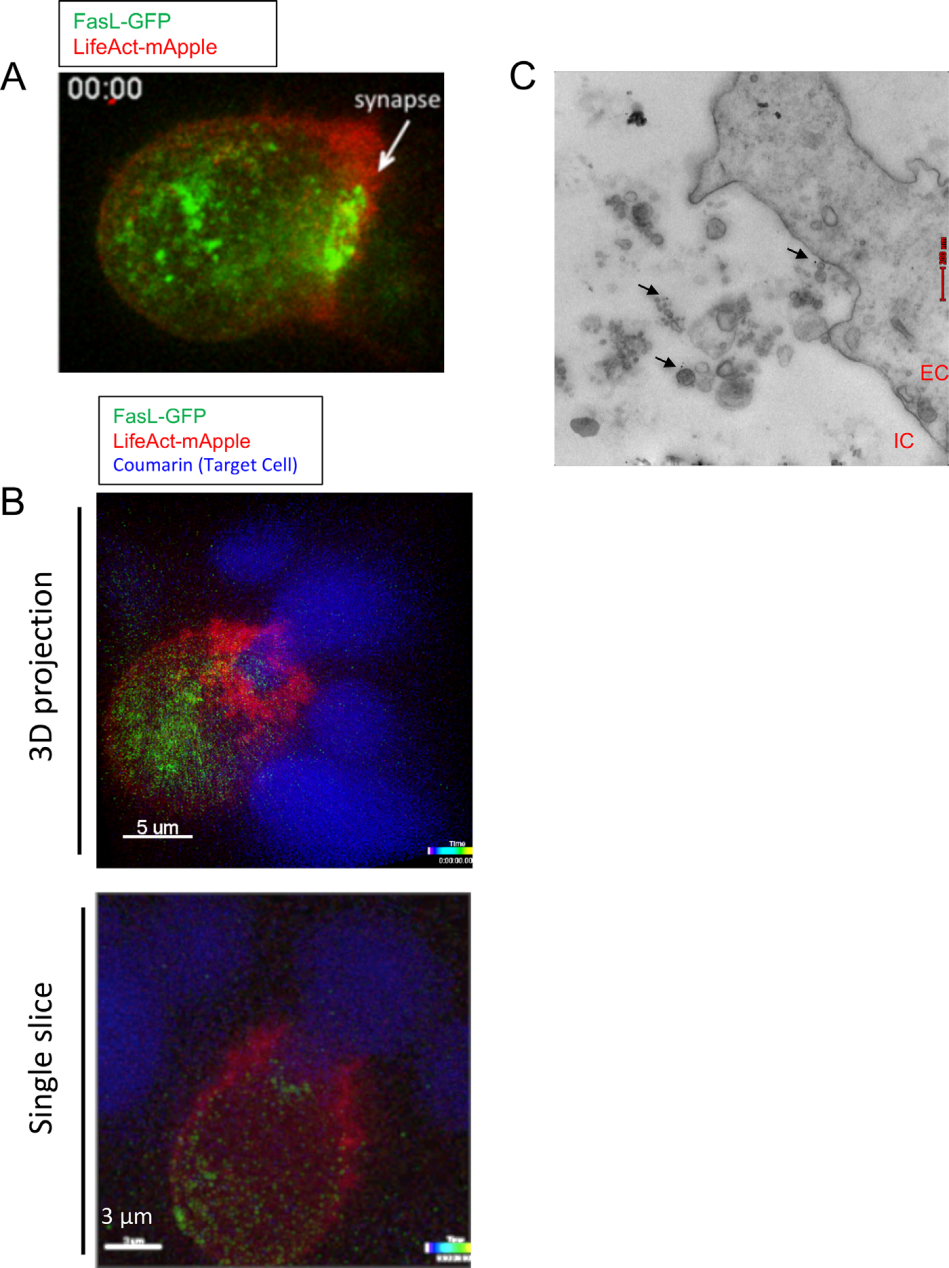
FasL localization to the immune synapse visualized during cell conjugate formation. (A) Still image from supplemental Video File 1, showing confocal immunofluorescence microscopy images of live YT cells stably expressing FasL‐GFP (green) transfected with a LifeAct‐mApple to label F‐actin (red). The image shows the time of maximal actin polymerization, which corresponds to FasL accumulation in the center of the immune synapse. (B) 3D projection and an image slice through the center of an NK:B cell conjugate showing the localization of FasL‐GFP (green) relative to Life‐Act (Red) and target cell nuclei (blue) (C) Immuno‐gold labeling of FasL in YT cells expressing FasL‐GFP treated with PMA and Ionomycin to induce degranulation. IC, Intracellular space; EC, Extracellular space, arrowheads mark FasL labeling.

To confirm FasL localization to the IS in with other markers of the immune synapse in living cells, we imaged NK‐cells transduced with FasL‐GFP forming conjugates with 721.221 B cells using live cell confocal video microscopy. FasL could be seen to localize to the immune synapse in the central area of the IS surrounded by F‐actin labeled by transfected LifeAct‐EGFP (Fig. 5A). The accompanying video (supplemental video 1) shows that FasL relocalizing away from the IS with similar kinetics to disassembly of F‐actin at the target cell interface. Imaging of FasL with CD63‐mCherry revealed colocalization of FasL and CD63 in the central portion of the NK‐B immune synapse (Fig. S3, supplemental video 2).

We asked whether FasL is released in vesicles upon degranulation by treating YT cells expressing FasL‐GFP with PMA and ionomycin before fixation and labeling. Electron micrographs showed that small vesicles released upon degranulation were labeled with FasL‐GFP, demonstrating that FasL is released in a membrane bound form upon secretion (Fig. 5C).

## Discussion

The transport and release of lytic proteins at the immunological synapse during target cell killing depends on the successful sorting of these proteins to secretory compartments. Human NK cells store lytic proteins such as perforin and granzymes in secretory lysosomes until signaled for degranulation 25. Our results identify CD63^+^ intraluminal vesicles (ILVs) within MVB as the primary subcellular compartment where FasL is localized. These results extend our earlier findings that FasL partially colocalized with perforin, cathepsin D, and granzyme A in NK cells 2. We now show that FasL is primarily localized to a broader population of CD63^+^ vesicles, with a smaller fraction of FasL colocalizing with LAMP1^+^and perforin^+^ mature secretory granules. The EM immunostaining revealed that FasL is localized to the ILVs of cytolytic granules, and is excluded from mature dense‐core vesicles where perforin and granzymes are localized 26, 27, 28, 29, concordant with the confocal microscopy results. Targeting sequences in the cytoplasmic N‐terminal of FasL likely direct FasL trafficking to a CD63+ secretory compartment, as in other cell types, FasL mutants lacking these sequences relocalize to the cell surface 21.

The localization of FasL to intraluminal vesicles of cytolytic granules is important as this will direct FasL to be released in its more potent, membrane‐bound form. Our results support earlier findings that FasL is found on exosomes 30, as ILVs will give rise to exosomes when released. In T cells, TCR activation induces colocalization of ADAM10 and FasL and cleavage of FasL into a soluble form 31, but it is not clear whether this occurs in NK cells. Although a significant portion of FasL‐containing vesicles traffic to the immune synapse (IS) during conjugate formation with target cells, in a majority of cells, a portion of FasL localizes to the distal pole of the NK cell away from the immune synapse. FasL at the immune synapse is likely involved in the target cell killing, confirming functional studies showing that FasL and perforin/granzyme mediated target cell killing are independent cytotoxic effectors in CD8+ cytotoxic T cells and NK cells 5, 32. The FasL at the distal pole of the cell may be in excess of the trafficking machinery needed to deliver FasL to the immune synapse, or may serve other functions. In T cells, the distal pole of the cell sequesters molecules such as CD43 away from the immune synapse and regulates T cell activation 33. The function of the distal pole complex in NK cells is less well understood, but FasL localized to the distal pole would be well‐positioned to induce cell death in bystander cells. In CD4^+^ T cells, FasL induced by restimulation through the TCR can kill other T cells not presenting antigen to the effector cells in an autocrine manner through FasL secreted in microvesicles 34. Since FasL‐GFP is expressed at supra‐physiological levels in these cell lines, this finding will need to be confirmed with endogenous FasL as well, although the localization of FasL to the immune synapse in NK cells is similar to what has been observed with native FasL in CD8^+^ T cells 2. As FasL trafficking is crucial for its function, these data provide additional information that may be useful for designing strategies to modulate the effects of FasL in relevant disease states.

## Materials and Methods

### Cell lines and culture

The human natural killer‐like (NK) cell line YT was cultured in RPMI‐1640 medium supplemented with 10% FCS, 100 U mL^−1^ rhIL‐2 (Proleukin), 2 mM L‐glutamine, 50 µM β‐mercaptoethanol, (100 U mL^−1^) penicillin/ (100 µg mL^−1^) streptomycin, and 1% sodium pyruvate (Gibco, Thermofisher Scientific, Waltham, MA, USA). The Epstein‐Barr Virus (EBV) transformed human B cell line, 721.221 was maintained in RPMI‐1640, supplemented with 10% FCS and (100 U mL^−1^) penicillin/ (100 µg mL^−1^) streptomycin.

### Lentiviral transduction of FasL‐GFP in YT cells

YT cells were transduced with GFP‐FasL using the lentiviral vector, pHR‐SINcPPT‐SGW as previously described (Stinchcombe et al., 2006). For each transduction experiment, 5 × 10^6^ YT cells were spun, pelleted, and resuspended in 1 mL of lentiviral supernatant in the presence of 6 µg mL^−1^ of polybrene (Sigma–Aldrich, Merck, Darmstadt, Germany) and plated in 24‐well plates. Cells were “spinfected” by centrifugation at 2500 rpm for 30 min at RT. Plates were then incubated at 37°C. About 24 h post‐infection viral supernatant was carefully removed from the top of the wells of infected YT cells and replaced with fresh complete YT medium. GFP‐FasL expression in YT cells was assayed by flow cytometry. Transduced YT cell clones were isolated by limiting dilution and selected based on GFP‐expression using flow cytometry.

### Confocal immunofluorescence microscopy

YT cells were resuspended at 10^6^ cells mL^−1^ in serum‐free RPMI‐1640 and adhered to glass slides for 30 min at 37°C, washed with PBS, containing 0.1% bovine serum albumin (BSA) (Sigma–Aldrich), 0.02% sodium azide and fixed with ice‐cold methanol for 5 min and washed in PBS before incubating for 1 h at room temperature in blocking buffer (PBS, 1% BSA). Samples were incubated with primary antibodies in PBS, 0.2% BSA for 1 h at room temperature, or overnight at 4°C and washed extensively in PBS, 0.2% BSA before adding secondary antibodies for 40 min at room temperature. Nuclei were stained with Hoechst 33342 (1:20,000) (Invitrogen, Thermofisher Scientific, Waltham, MA, USA) in PBS for 1–2 min before mounting with number 1.5 coverglass and mounting medium (Mowiol). Fixed images were examined at room temperature using either Zeiss LSM510 confocal microscope (Carl Zeiss, Inc., Oberkochen, Germany), or with laser‐scanning spinning‐disk confocal system (Andor Revolution) with lasers exciting at 405, 488, 543, and 633 nm using the 63x or 100x (Plan‐Apochromat, NA 1.40) oil immersion objective. Images were acquired using Image Pro (Zeiss, Inc.) or IQ software (Andor, Belfast, Northern Ireland).

### Colocalization analysis

Imaris Scientific Image Processing and Analysis Software (BitPlane Scientific Software, Zurich, Switzerland) was used for colocalization analysis. Each image stack contained several cells, with 76 cells (12 stacks) analyzed for GFP‐FasL to perforin colocalization, 78 cells (14 stacks) for GFP‐FasL to LAMP1 colocalization, 31 cells (7 stacks) for GFP‐FasL to CD63 colocalization and 237 cells (35 stacks) for GFP‐FasL to anti‐GFP antibody colocalization. GFP‐FasL was displayed as channel A. Anti‐perforin, anti‐LAMP1, anti‐CD63 (Alexa‐Fluor 568‐coupled secondary antibody) or anti‐GFP (Alex Fluor 533‐coupled secondary antibody) staining was displayed as channel B. Channel thresholds were set as to include the full range of data as displayed in the colocalization tool 2D histogram. For quantitative colocalization analysis, each image stack was analyzed by creating a mask via the GFP‐FasL channel (threshold 600–750) that focuses the region of interest (ROI) for colocalization analysis to the FasL+ structures. Pearson's coefficients for each mask (ROI) were plotted using Prism software and statistical analysis was done using the one‐way ANOVA test (parametric, (*F*(6,94) = 153.6, *p* < 0.0001)) plus Bonferroni's Multiple Comparison post hoc test (95% confidence intervals).

### NK‐target cell conjugate formation

Equal volumes of GFP‐WT FasL‐expressing YT clones and 721.221 B cells were resuspended at a concentration of 0.75–.0.80 × 10^6^ cells mL^−1^ in serum‐free medium. Prior to this final working concentration, all cells were washed in complete RPMI‐1640 medium. A total volume of 300 µL of target cells was plated on a 35 mm petri dish (MatTek Corporation) for 5 min at 37°C in RPMI‐1640. A 1.5 mL of Imaging Buffer which was prepared in RPMI‐1640 (no phenol red), supplemented with 10% FCS, 25 mM HEPES, Penicillin/Streptomycin, and L‐glutamine, was added to the plated cells. YT cells were spun down in Imaging Buffer for 2 min at RT, resuspended and then added on top of target cells in the petri dish. Cells were fixed at the indicated times and imaged by confocal miscroscopy as described above.

### Electron microscopy

Cells were fixed with 4% PFA, 0.2% glutaraldehyde/ PBS. Cells were washed with 15 mM glycine and scraped and pelleted in 12% gelatin. Pellets were cut out and infiltrated with 2.3M sucrose before being mounted onto pins. Ultrathin cryosections were cut at −120°C using a Leica Ultracut UCT microtome. Sections were picked up with 2.3 M sucrose. Sections were labeled with a rabbit polyclonal anti‐GFP antibody (Abcam, Cambridge, UK—ab6556) and 10 nm Protein‐A gold. Sections were visualised with a FEI Tecnai TEM at 80 kV.

## Conflict of Interest

None declared.

## Supporting information

Additional supporting information may be found in the online version of this article at the publisher's web‐site.

**Figure S1**. Validation of GFP as a marker for FasL subcellular localization. Confocal immunofluorescence microscopy of YT cells stably expressing wild‐type FasL‐GFP fusion protein (green) additionally stained with antibodies to either FasL (Nok1 antibody, red, A) or GFP (anti‐GFP antibody, red, B). The nuclei of YT cells were labeled using Hoechst dye.
**Figure S2**. Line plot analysis of FasL colocalization with secretory lysosome markers. Confocal immunofluorescence microscopy (slices) of YT cells stably expressing wild‐type FasL (green) were stained with anti‐LAMP1 antibody to label LAMP 1+ compartments (B), anti‐CD63 antibody to label CD63+ compartments (C), and with anti‐perforin antibody (δG9) to label perforin‐containing compartments (A). The nuclei of YT cells were labeled with Hoechst dye. Merge1 is GFP‐FasL (green), Hoechst (blue), and the vesicle of interest (LAMP1, CD63, perforin, red). Merge2 is GFP‐FasL (green), Hoechst (blue), and anti‐GFP (White). The lines in the graph (D) represent the presence of FasL (green) and LAMP1, CD63 or perforin marker (red), and Hoechst staining (blue), and anti‐GFP (black) going across the line shown in the FasL panel.
**Figure S3**. Colocalization of FasL and CD63 at the immune synapse in living cells. YT cell clones that express GFP‐FasL (green) and were transfected with mCherry‐CD63 to express CD63 (in red). Target cells (blue) (721.221 B cell line) were loaded with Coumarin‐blue dye and then added to YT cells. Each picture is a merge of blue, red and green confocal immunofluorescence images. The individual pictures making up the montage were taken from live cell time‐lapse video microscopy. Images were acquired‘ from 0 to 12 min after YT and B cells came into contact.

**Supplemental Video 1, accompanying Figure 5A** Supplemental Video 1, accompanying Figure 5A. YT cell clones that express GFP‐FasL (green) were transfected with LifeAct‐apple plasmid to track F‐actin (red). Target cells (721.221 B cell line) were added to YT cells. The video is a merge of red and green confocal immunofluorescence images. This live cell time‐lapse video was taken from 0 to 12 min after YT and B cells came into contact. The video plays at 100× real‐time speed (40 frames acquired at 20 s intervals played at 5 frames per second).

**Supplemental Video 2, accompanying Figure S3** Supplemental Video 2, accompanying Figure S3. YT cell clones that express GFP‐FasL (green) and were transfected with mCherry‐CD63 to express CD63 (in red). Target cells (blue) (721.221 B cell line) were loaded with Coumarin‐blue dye and then added to YT cells. The video is a merge of the red, green and blue confocal immunofluorescence images. This live cell time‐lapse video was taken from 0 to 12 min after YT and B cells came into contact. The video plays at 100× real‐time speed (40 frames acquired at 20 s intervals played at 5 frames per second).

## References

[1] O' Reilly, L. A. , L. Tai , L. Lee , E. A. Kruse , S. Grabow , W. D. Fairlie , N. M. Haynes , D. M. Tarlinton , J. G. Zhang , G. T. Belz , et al. 2009 Membrane‐bound Fas ligand only is essential for Fas‐induced apoptosis. Nature 461:659–663. 19794494 10.1038/nature08402 PMC2785124

[2] Bossi, G. , and G. M. Griffiths . 1999 Degranulation plays an essential part in regulating cell surface expression of Fas ligand in T cells and natural killer cells. Nat. Med. 5:90–96. 9883845 10.1038/4779

[3] Kojima, Y. , A. Kawasaki‐Koyanagi , N. Sueyoshi , A. Kanai , H. Yagita , and K. Okumura . 2002 Localization of Fas ligand in cytoplasmic granules of CD8+ cytotoxic T lymphocytes and natural killer cells: participation of Fas ligand in granule exocytosis model of cytotoxicity. Biochem. Biophys. Res. Commun. 296:328–336. 12163021 10.1016/s0006-291x(02)00841-0

[4] Lowin, B. , F. Beermann , A. Schmidt , and J. Tschopp . 1994 A null mutation in the perforin gene impairs cytolytic T lymphocyte‐ and natural killer cell‐mediated cytotoxicity. Proc. Natl. Acad. Sci. U. S. A. 91:11571–11575. 7972104 10.1073/pnas.91.24.11571 PMC45273

[5] Oshimi, Y. , S. Oda , Y. Honda , S. Nagata , and S. Miyazaki . 1996 Involvement of Fas ligand and Fas‐mediated pathway in the cytotoxicity of human natural killer cells. J. Immunol. 157:2909–2915. 8816396

[6] Stranges, P. B. , J. Watson , C. J. Cooper , C. M. Choisy‐Rossi , A. C. Stonebraker , R. A. Beighton , H. Hartig , J. P. Sundberg , S. Servick , G. Kaufmann , et al. 2007 Elimination of antigen‐presenting cells and autoreactive T cells by Fas contributes to prevention of autoimmunity. Immunity 26:629–641. 17509906 10.1016/j.immuni.2007.03.016 PMC2575811

[7] Hao, Z. , G. S. Duncan , J. Seagal , Y. W. Su , C. Hong , J. Haight , N. J. Chen , A. Elia , A. Wakeham , W. Y. Li , et al. 2008 Fas receptor expression in germinal‐center B cells is essential for T and B lymphocyte homeostasis. Immunity 29:615–627. 18835195 10.1016/j.immuni.2008.07.016 PMC3470429

[8] Straus, S. E. , E. S. Jaffe , J. M. Puck , J. K. Dale , K. B. Elkon , A. Rosen‐Wolff , A. M. Peters , M. C. Sneller , C. W. Hallahan , J. Wang , et al. 2001 The development of lymphomas in families with autoimmune lymphoproliferative syndrome with germline Fas mutations and defective lymphocyte apoptosis. Blood 98:194–200. 11418480 10.1182/blood.v98.1.194

[9] Magerus‐Chatinet, A. , M. C. Stolzenberg , N. Lanzarotti , B. Neven , C. Daussy , C. Picard , N. Neveux , M. Desai , M. Rao , K. Ghosh , et al. 2013 Autoimmune lymphoproliferative syndrome caused by a homozygous null FAS ligand (FASLG) mutation. J. Allergy Clin. Immunol. 131:486–490. 22857792 10.1016/j.jaci.2012.06.011 PMC3824280

[10] Hughes, P. D. , G. T. Belz , K. A. Fortner , R. C. Budd , A. Strasser , and P. Bouillet . 2008 Apoptosis regulators Fas and Bim cooperate in shutdown of chronic immune responses and prevention of autoimmunity. Immunity 28:197–205. 18275830 10.1016/j.immuni.2007.12.017 PMC2270348

[11] Abougergi, M. S. , S. J. Gidner , D. K. Spady , B. C. Miller , and D. L. Thiele . 2005 Fas and TNFR1, but not cytolytic granule‐dependent mechanisms, mediate clearance of murine liver adenoviral infection. Hepatology 41:97–105. 15619234 10.1002/hep.20504 PMC2666068

[12] Fleck, M. , E. R. Kern , T. Zhou , J. Podlech , W. Wintersberger , C. K. Edwards 3rd , and J. D. Mountz . 1998 Apoptosis mediated by Fas but not tumor necrosis factor receptor 1 prevents chronic disease in mice infected with murine cytomegalovirus. J. Clin. Invest. 102:1431–1443. 9769336 10.1172/JCI3248 PMC508991

[13] Kirkin, V. , N. Cahuzac , F. Guardiola‐Serrano , S. Huault , K. Luckerath , E. Friedmann , N. Novac , W. S. Wels , B. Martoglio , A. O. Hueber , et al. 2007 The Fas ligand intracellular domain is released by ADAM10 and SPPL2a cleavage in T‐cells. Cell Death Differ. 14:1678–1687. 17557115 10.1038/sj.cdd.4402175

[14] Reilly, O. , L. A. Tai , L. Lee , L. Kruse , E. A. Grabow , S. Fairlie , W. D. Haynes , N. M. Tarlinton , D. M. Zhang , J. G. Belz , et al. 2009 Membrane‐bound Fas ligand only is essential for Fas‐induced apoptosis. Nature 461:659–663. 19794494 10.1038/nature08402 PMC2785124

[15] Suda, T. 1995 Expression of the Fas ligand in cells of T cell lineage. J. Immunol 154:3806–3813. 7706720

[16] Kagi, D. , F. Vignaux , B. Ledermann , K. Burki , V. Depraetere , S. Nagata , H. Hengartner , and P. Golstein . 1994 Fas and perforin pathways as major mechanisms of T cell‐mediated cytotoxicity. Science 265:528–530. 7518614 10.1126/science.7518614

[17] Jodo, S. , S. Xiao , A. Hohlbaum , D. Strehlow , A. Marshak‐Rothstein , and S. T. Ju . 2001 Apoptosis‐inducing membrane vesicles. A novel agent with unique properties. J. Biol. Chem. 276:39938–39944. 11546786 10.1074/jbc.M107005200

[18] Martinez‐Lorenzo, M. J. , A. Anel , S. Gamen , I. Monle , P. Lasierra , L. Larrad , A. Pineiro , M. A. Alava , and J. Naval . 1999 Activated human T cells release bioactive Fas ligand and APO2 ligand in microvesicles. J. Immunol. 163:1274–1281. 10415024

[19] Frängsmyr, L. , V. Baranov , O. Nagaeva , U. Stendahl , L. Kjellberg , and L. Mincheva‐Nilsson . 2005 Cytoplasmic microvesicular form of Fas ligand in human early placenta: switching the tissue immune privilege hypothesis from cellular to vesicular level. Mol. Hum. Reprod. 11:35–41. 15579659 10.1093/molehr/gah129

[20] Monleon, I. , M. J. Martinez‐Lorenzo , L. Monteagudo , P. Lasierra , M. Taules , M. Iturralde , A. Pineiro , L. Larrad , M. A. Alava , J. Naval , et al. 2001 Differential secretion of Fas ligand‐ or APO2 ligand/TNF‐related apoptosis‐inducing ligand‐carrying microvesicles during activation‐induced death of human T cells. J. Immunol. 167:6736–6744. 11739488 10.4049/jimmunol.167.12.6736

[21] Blott, E. J. , G. Bossi , R. Clark , M. Zvelebil , and G. M. Griffiths . 2001 Fas ligand is targeted to secretory lysosomes via a proline‐rich domain in its cytoplasmic tail. J. Cell Sci. 114:2405–2416. 11559749 10.1242/jcs.114.13.2405

[22] Takahashi, T. , M. Tanaka , C. I. Brannan , N. A. Jenkins , N. G. Copeland , T. Suda , and S. Nagata . 1994 Generalized lymphoproliferative disease in mice, caused by a point mutation in the Fas ligand. Cell 76:969–976. 7511063 10.1016/0092-8674(94)90375-1

[23] Lynch, D. H. , M. L. Watson , M. R. Alderson , P. R. Baum , R. E. Miller , T. Tough , M. Gibson , T. Davissmith , C. A. Smith , K. Hunter , et al. 1994 The mouse fas‐ligand gene is mutated in gld mice and is part of a tnf family gene‐Cluster. Immunity 1:131–136. 7889405 10.1016/1074-7613(94)90106-6

[24] Ramsdell, F. , M. S. Seaman , R. E. Miller , T. W. Tough , M. R. Alderson , and D. Lynch . 1994 H., gld/gld mice are unable to express a functional ligand for Fas. Eur. J. Immunol. 24:928–933. 7512035 10.1002/eji.1830240422

[25] Smyth, M. J. , and J. A. Trapani . 1995 Granzymes: exogenous proteinases that induce target cell apoptosis. Immunol. Today 16:202–206. 7734049 10.1016/0167-5699(95)80122-7

[26] Peters, P. J. , H. J. Geuze , H. A. Van der Donk , J. W. Slot , J. M. Griffith , N. J. Stam , H. C. Clevers , and J. Borst . 1989 Molecules relevant for T cell‐target cell interaction are present in cytolytic granules of human T lymphocytes. Eur. J. Immunol. 19:1469–1475. 2789142 10.1002/eji.1830190819

[27] Peters, P. J. , J. Borst , V. Oorschot , M. Fukuda , O. Krahenbuhl , J. Tschopp , J. W. Slot , and H. J. C. Geuze . 1991 T lymphocyte granules are secretory lysosomes, containing both perforin and granzymes. J. Exp. Med. 173:1099–1109. 2022921 10.1084/jem.173.5.1099 PMC2118839

[28] Burkhardt, J. K. , S. Hester , C. K. Lapham , and Y. Argon . 1990 The lytic granules of natural killer cells are dual‐function organelles combining secretory and pre‐lysosomal compartments. J. Cell Biol. 111:2327–2340. 2277062 10.1083/jcb.111.6.2327 PMC2116378

[29] Burkhardt, J. K. , S. Hester , and Y. Argon . 1989 Two proteins targeted to the same lytic granule compartment undergo very different posttranslational processing. Proc. Natl. Acad. Sci. U. S. A. 86:7128–7132. 2674947 10.1073/pnas.86.18.7128 PMC298008

[30] Alonso, R. , M. C. Rodriguez , J. Pindado , E. Merino , I. Merida , and M. Izquierdo . 2005 Diacylglycerol kinase alpha regulates the secretion of lethal exosomes bearing Fas ligand during activation‐induced cell death of T lymphocytes. J. Biol. Chem. 280:28439–28450. 15870081 10.1074/jbc.M501112200

[31] Ebsen, H. , M. Lettau , D. Kabelitz , and O. Janssen . 2015 Subcellular localization and activation of ADAM proteases in the context of FasL shedding in T lymphocytes. Mol. Immunol. 65:416–428. 25745808 10.1016/j.molimm.2015.02.008

[32] Zamai, L. , M. Ahmad , I. M. Bennett , L. Azzoni , E. S. Alnemri , and B. Perussia . 1998 Natural killer (NK) cell‐mediated cytotoxicity: differential use of TRAIL and Fas ligand by immature and mature primary human NK cells. J. Exp. Med. 188:2375–2380. 9858524 10.1084/jem.188.12.2375 PMC2212426

[33] Cullinan, P. , A. I. Sperling , and J. K. Burkhardt . 2002 The distal pole complex: a novel membrane domain distal to the immunological synapse. Immunol. Rev. 189:111–122. 12445269 10.1034/j.1600-065x.2002.18910.x

[34] Martinez‐Lorenzo, M. J. , A. Anel , S. Gamen , I. Monle , P. Lasierra , L. Larrad , A. Pineiro , M. A. Alava , and J. Naval . 1999 Activated human T cells release bioactive Fas ligand and APO2 ligand in microvesicles. J. Immunol. 163:1274–1281. 10415024

