# Supplementary figures and images for: Fas Ligand localizes to intraluminal vesicles within NK cell cytolytic granules and is enriched at the immune synapse
Source: Immun Inflamm Dis. 2018 Apr 11;6(2):312–21. doi: 10.1002/iid3.219 (PMC5946154; doi:10.1002/iid3.219)

Figure S1

A

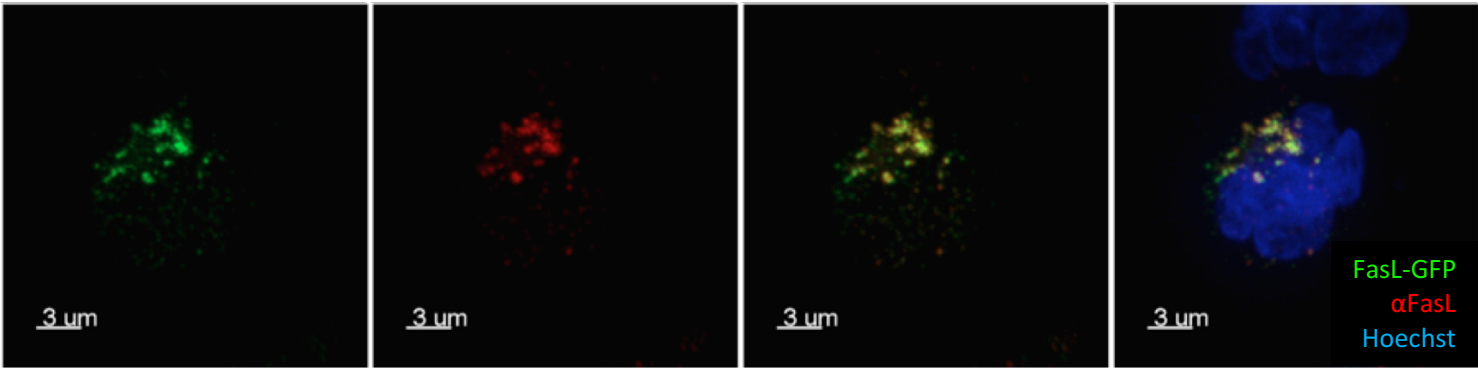

B

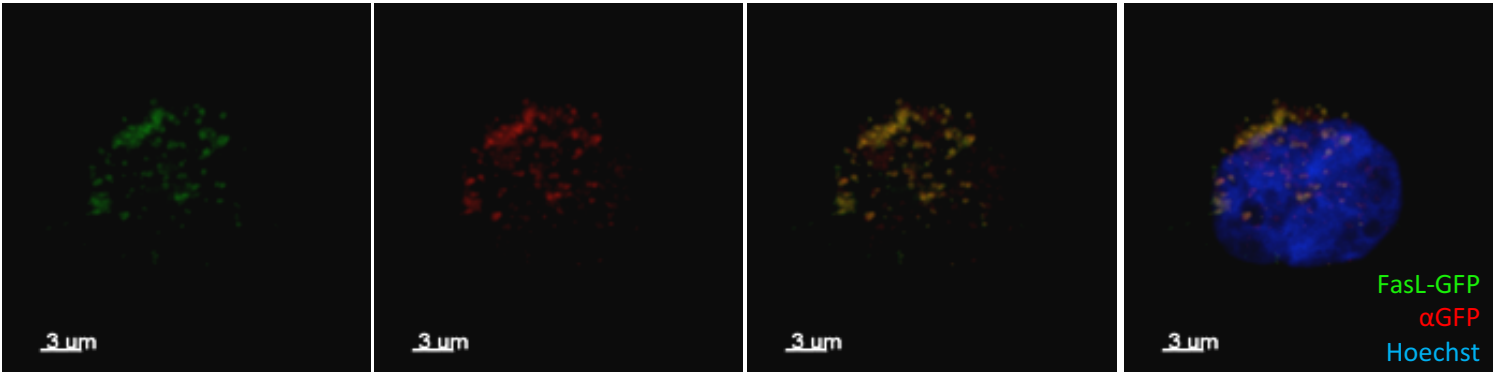

Figure S2

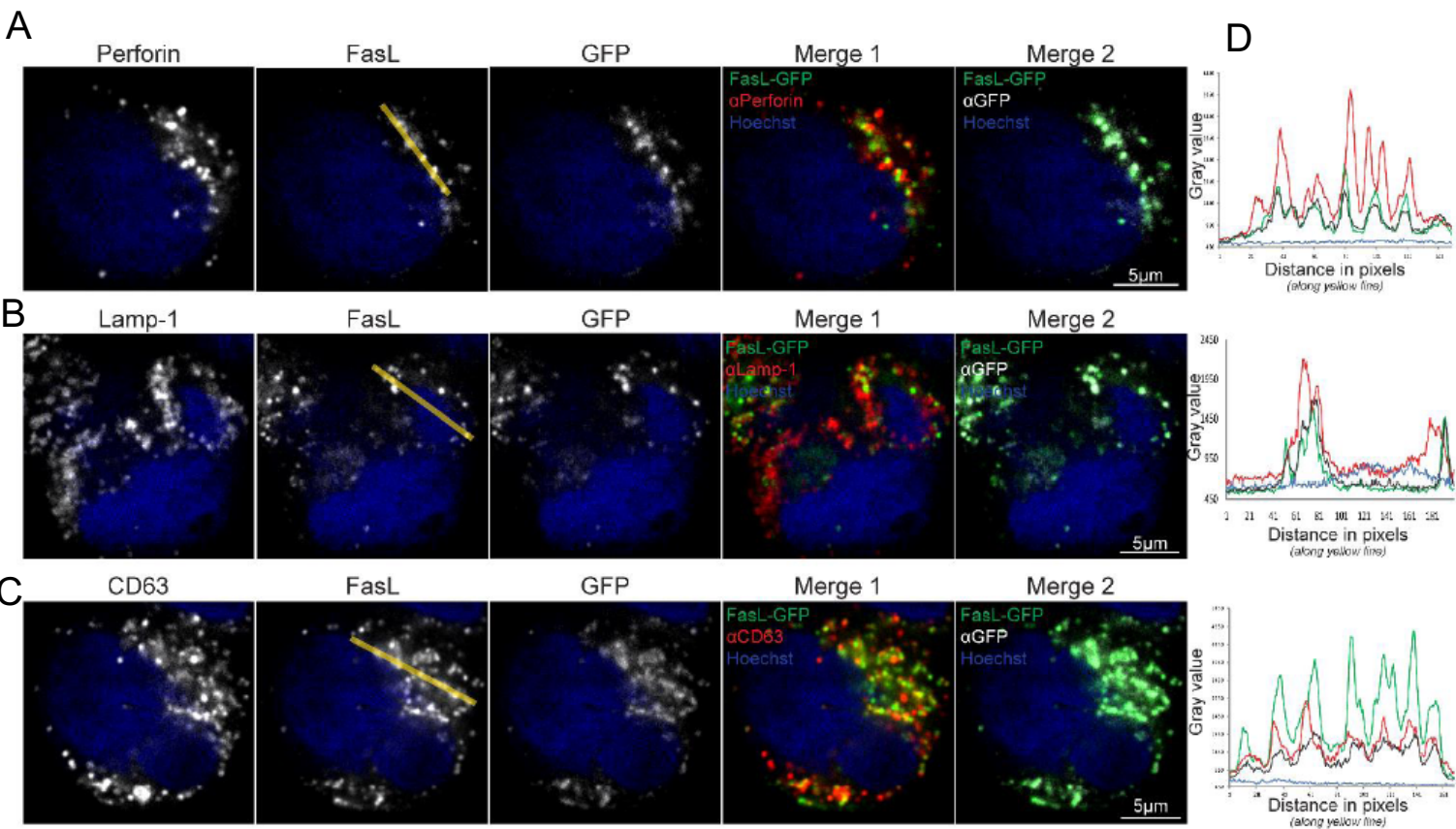

Figure S3

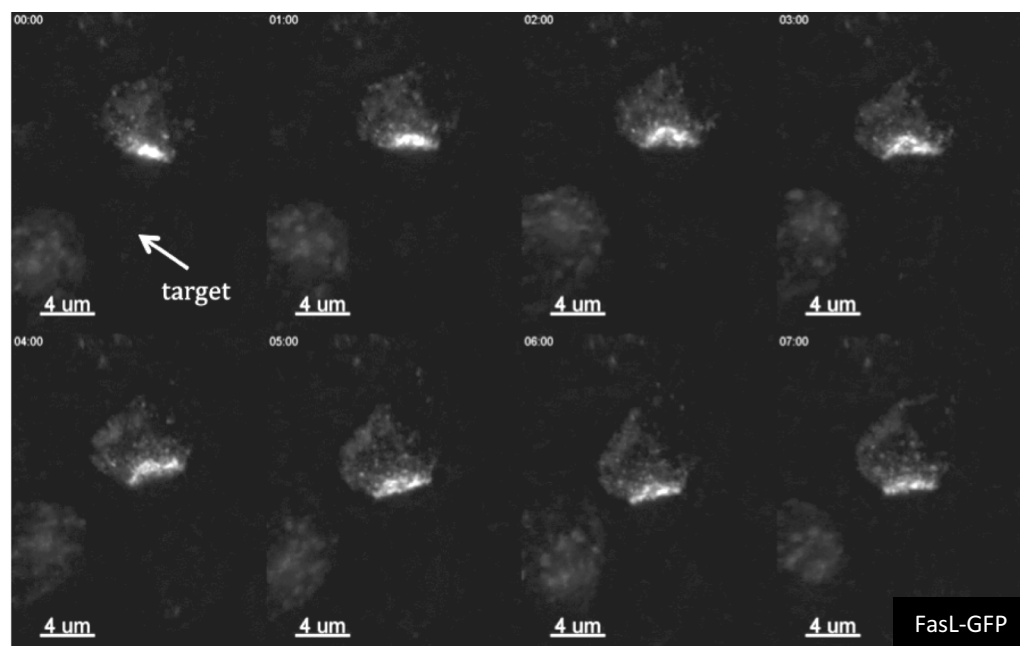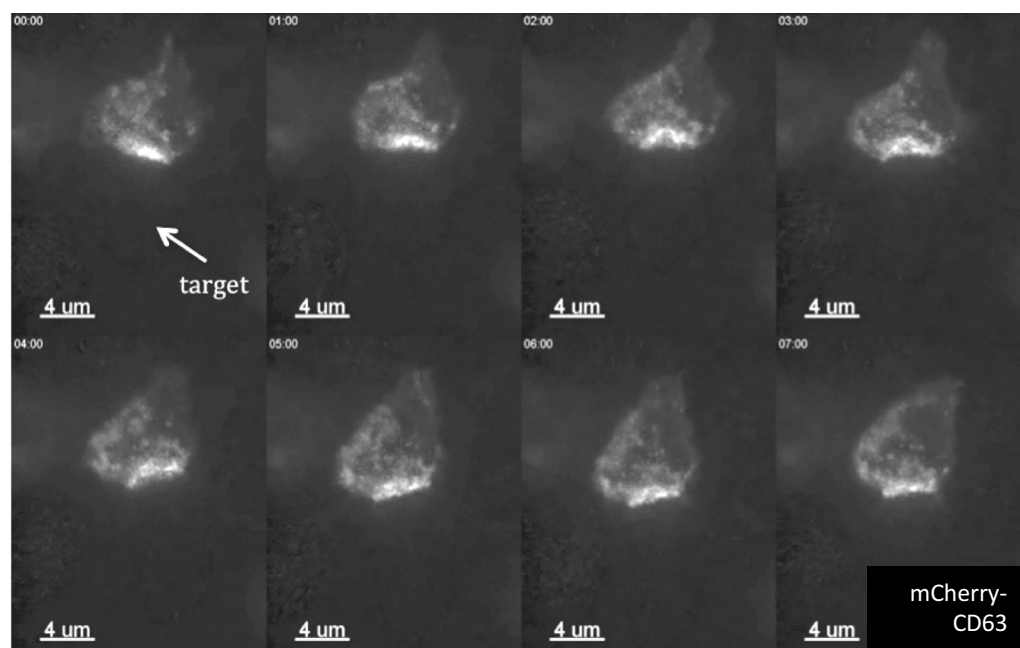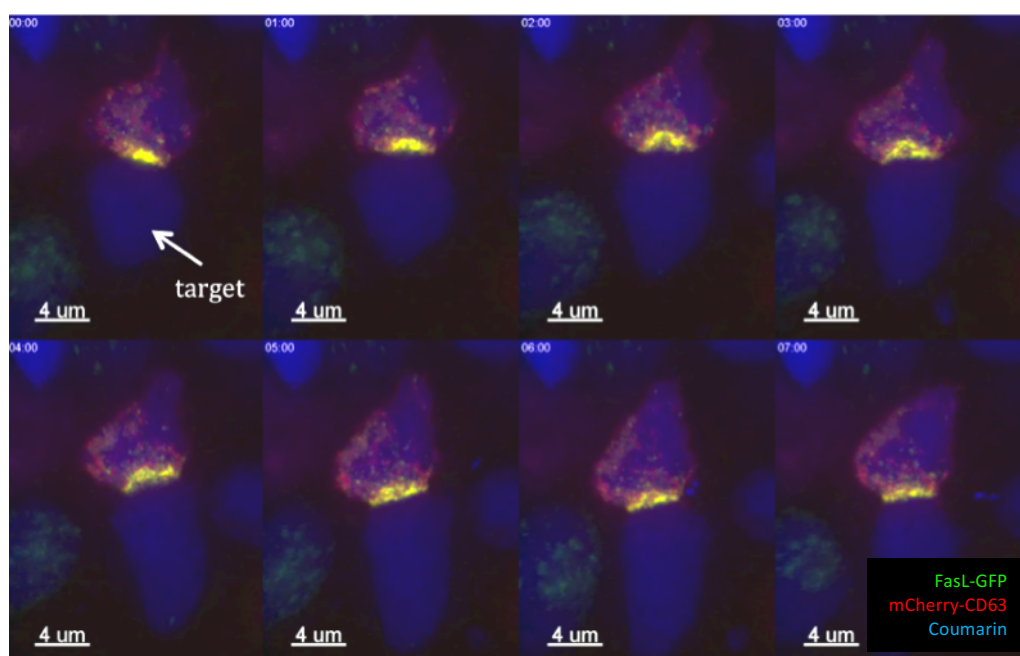

Supplement: Supplementary file 1 — Figure S1. Validation of GFP as a marker for FasL subcellular localization. Confocal immunofluorescence microscopy of YT cells stably expressing wild‐type FasL‐GFP fusion protein (green) additionally stained with antibodies to either FasL (Nok1 antibody, red, A) or GFP (anti‐GFP antibody, red, B). The nuclei of YT cells were labeled using Hoechst dye. Figure S2. Line plot analysis of FasL colocalization with secretory lysosome markers. Confocal immunofluorescence microscopy (slices) of YT cells stably expressing wild‐type FasL (green) were stained with anti‐LAMP1 antibody to label LAMP 1+ compartments (B), anti‐CD63 antibody to label CD63+ compartments (C), and with anti‐perforin antibody (δG9) to label perforin‐containing compartments (A). The nuclei of YT cells were labeled with Hoechst dye. Merge1 is GFP‐FasL (green), Hoechst (blue), and the vesicle of interest (LAMP1, CD63, perforin, red). Merge2 is GFP‐FasL (green), Hoechst (blue), and anti‐GFP (White). The lines in the graph (D) represent the presence of FasL (green) and LAMP1, CD63 or perforin marker (red), and Hoechst staining (blue), and anti‐GFP (black) going across the line shown in the FasL panel. Figure S3. Colocalization of FasL and CD63 at the immune synapse in living cells. YT cell clones that express GFP‐FasL (green) and were transfected with mCherry‐CD63 to express CD63 (in red). Target cells (blue) (721.221 B cell line) were loaded with Coumarin‐blue dye and then added to YT cells. Each picture is a merge of blue, red and green confocal immunofluorescence images. The individual pictures making up the montage were taken from live cell time‐lapse video microscopy. Images were acquired‘ from 0 to 12 min after YT and B cells came into contact. [file IID3-6-312-s001.pdf]
